# Treatment of Two Canals in All Mandibular Incisor Teeth in the Same Patient

**DOI:** 10.1155/2014/893980

**Published:** 2014-11-16

**Authors:** Vandana B. Kokane, Swapnil N. Patil, Mohit K. Gunwal, Rajesh Kubde, Swaraj Atre

**Affiliations:** Department of Conservative Dentistry & Endodontics, VSPM Dental College & Research Centre of Nagpur, Maharashtra University of Health Sciences, Nashik 440019, India

## Abstract

The main reason for unfavourable outcome in endodontic treatment of mandibular incisor is the inability to detect the presence of second canal. Pain even after extirpation of complete pulp tissue from root canal of vital teeth is the main indication of hidden canals. The present case report is also on pain because of another neglected canal in all mandibular incisors in the same patient.

## 1. Introduction

The main reason for the failure of endodontic therapy is incomplete knowledge about the anatomical variation of root canals. A canal is often left untreated because the dentist fails to recognize its presence either due to lack of knowledge of root canal morphology or due to lack of experience and skill to negotiate the canal. The dentist must have a thorough knowledge of root canal morphology before starting endodontic treatment. For good prognosis following root canal treatment, the entire root canal system must be explored, adequately debrided, and filled. Therefore, clinicians must be familiar with the various root canal configurations [1].

Various authors have studied the root canal morphology of extracted mandibular incisors and have reported a prevalence of two canals in 12–35% of the cases. However, the prevalence of multiple canals in all mandibular incisors of the same patients has not been reported [1].

This case report presents the endodontic treatment of all mandibular incisors, each having two separate canals which merge into a single canal before exiting the tooth through a single apical foramen [2–4].

## 2. Case Description

**Figure 1:**
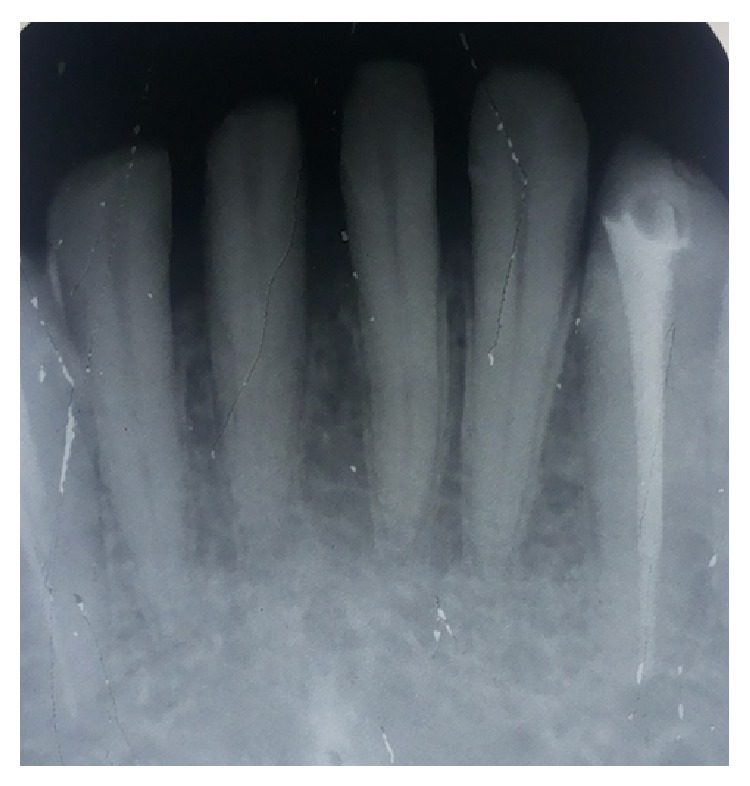
Preoperative radiograph.

A 47-year-old lady reported to the Department of Conservative Dentistry and Endodontics of VSPM Dental College and Research Centre of Nagpur with chief complaint of pain in lower anterior teeth. On clinical examination, 31, 32, 41, and 42 were tender on vertical percussion. Restoration was evident with respect to 31, 32, 41, and 42 which is suggestive of prior dental treatment from private clinic with no relief from symptoms. While taking dental history, patient reveals the story that she had visited private dental clinic for pain in 31, 32, 41, and 42 where she had undergone some dental treatment with all lower anterior teeth. But, even after several visits, there was no relief from pain in 31, 32, 41, and 42. Radiographic examination revealed prior endodontic therapy with obturation in 33, 43, and 44 and access opening procedure with 31, 32, 41, and 42. Two root canals were observed in 41 (Figure 1). A possibility of missed canals was suspected and the treatment for 31, 32, 41, and 42 was planned accordingly.

**Figure 2:**
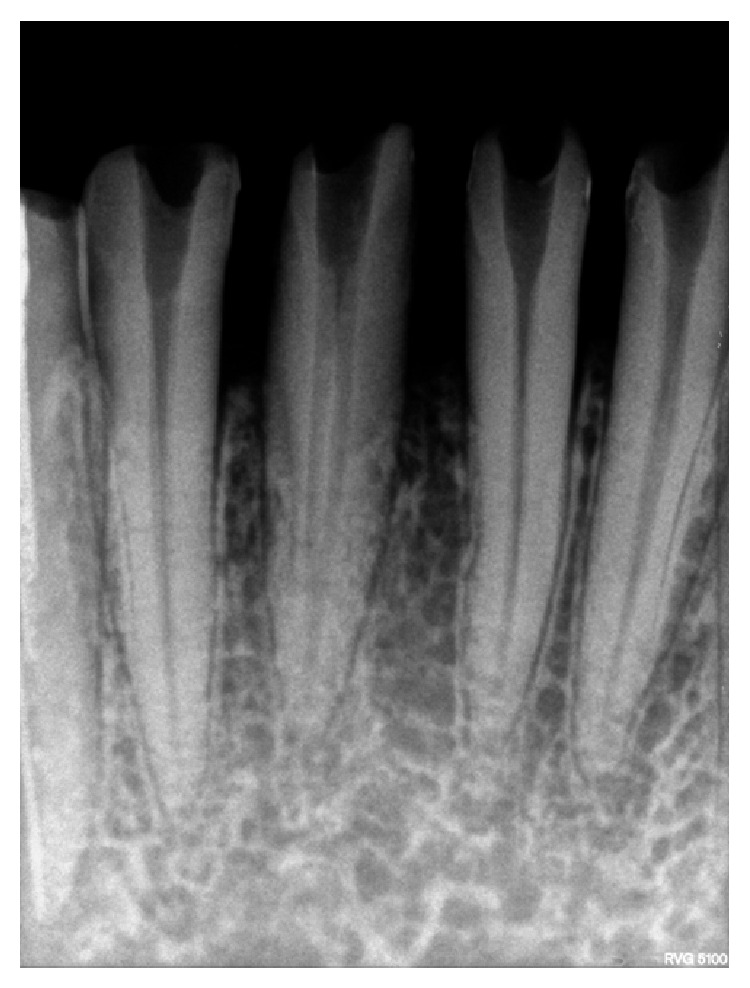
Radiograph showing modified access opening.

**Figure 3:**
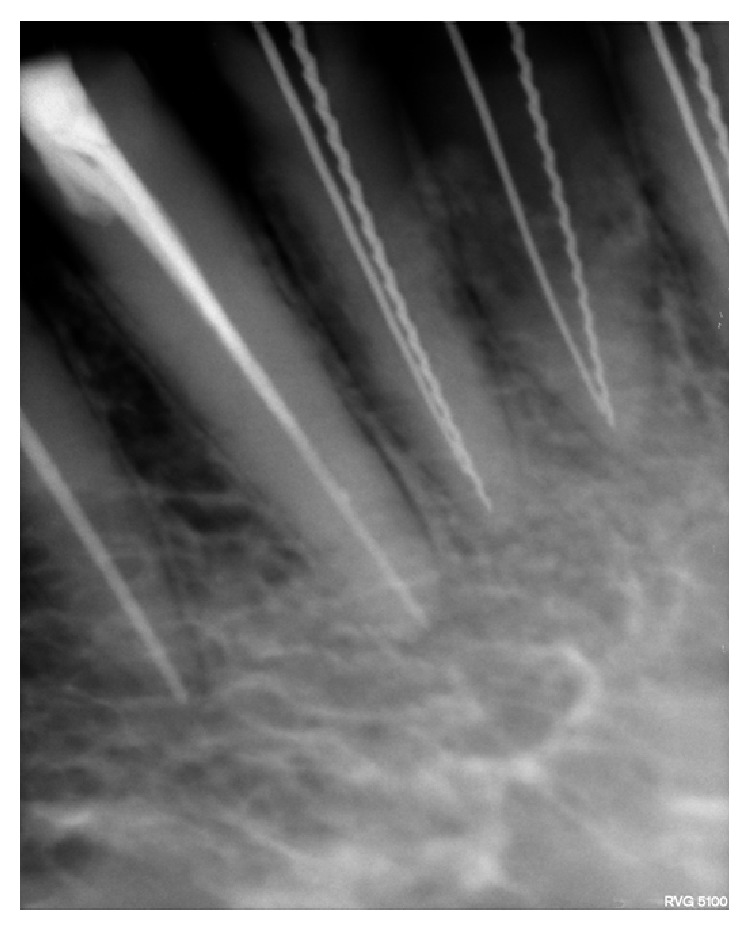
Radiograph showing two different instruments (“H” file and Reamer) in two canals in 31 and 32.

**Figure 4:**
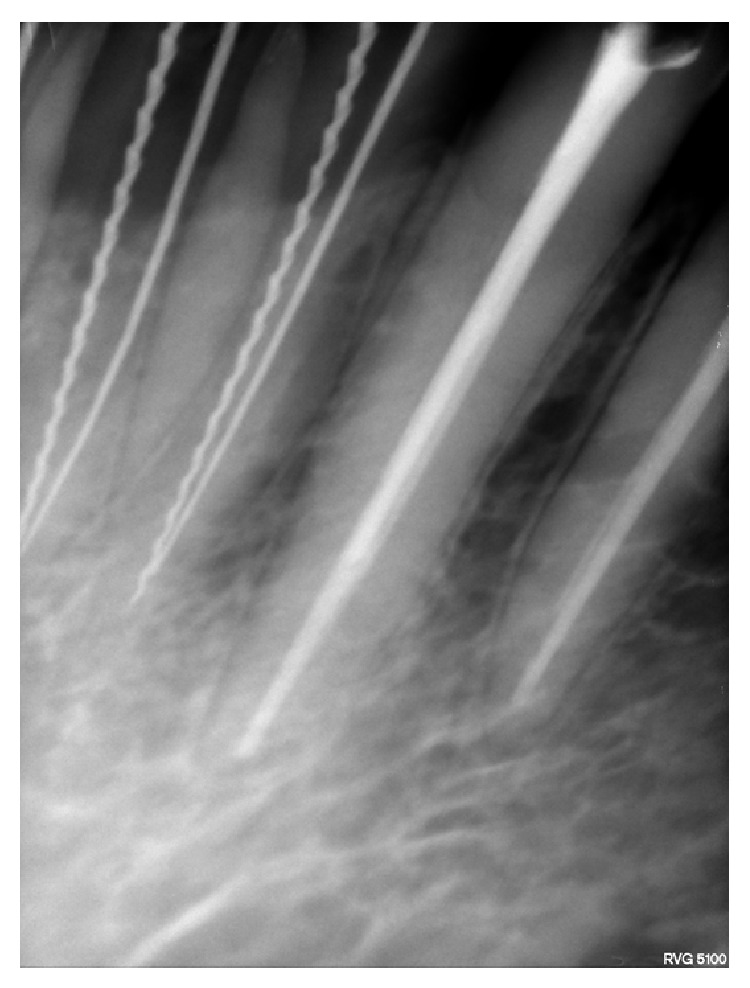
Radiograph showing two different instruments (“H” file and Reamer) in two canals in 41 and 42.

**Figure 5:**
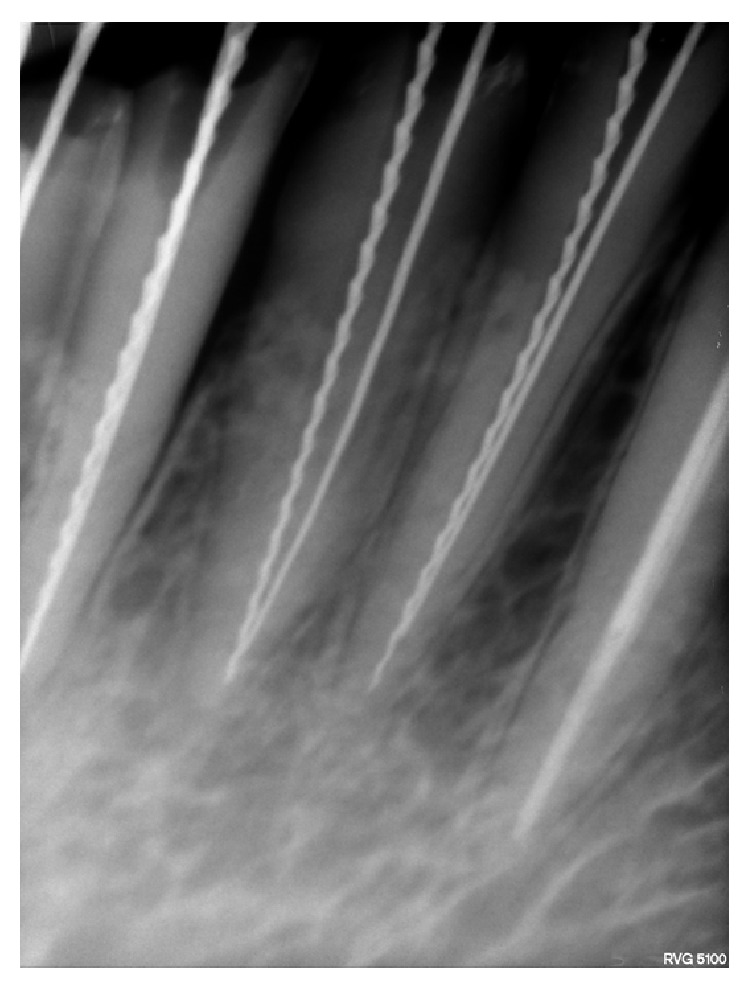
Radiograph showing two different instruments (“H” file and Reamer) in two canals in 41 and 42.

The access openings of 31, 32, 41, and 42 were modified and widened buccolingually and extended into cingulum gingivally, which revealed the presence of a lingual canal (Figure 2). The patency was checked using a no. 10 k file. Working length was determined by placing a 20. no. H file in the buccal canal & 15 no. reamer in the lingual canal using digital radiography. The presence of separate canals was confirmed using different angulations (Figures 3, 4, and 5). Biomechanical preparation was carried out using conventional hand instruments. 2.5% of sodium hypochlorite and 17% EDTA were used for irrigation. The canals were rinsed with normal saline after each instrument change.

**Figure 6:**
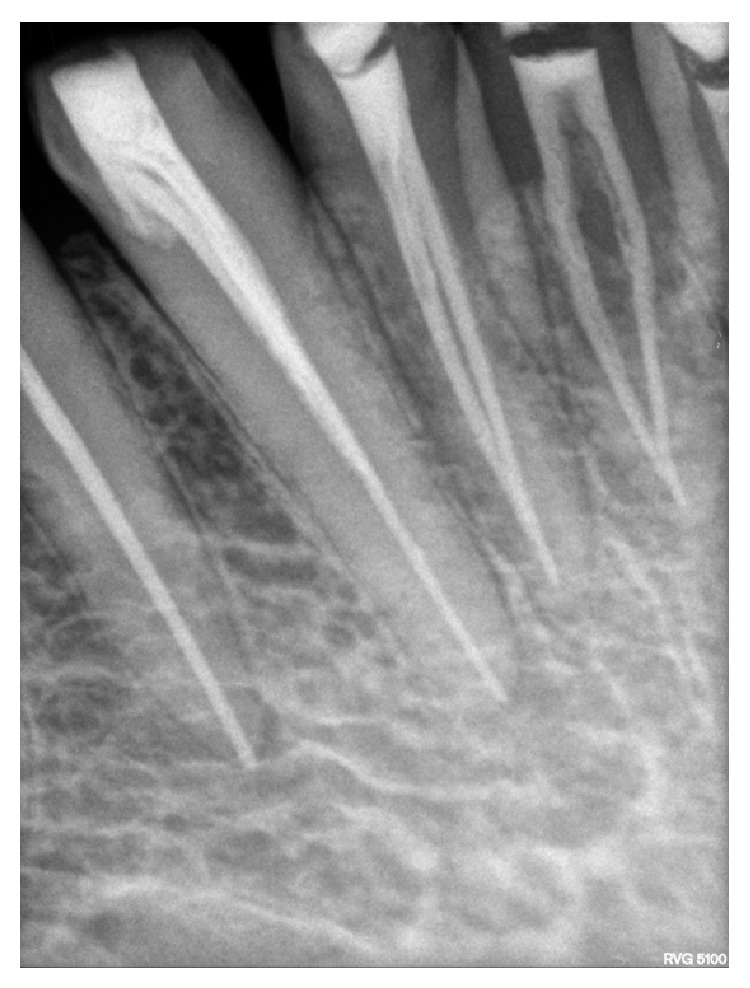
Radiograph showing obturation in two separate canals in 31 and 32.

**Figure 7:**
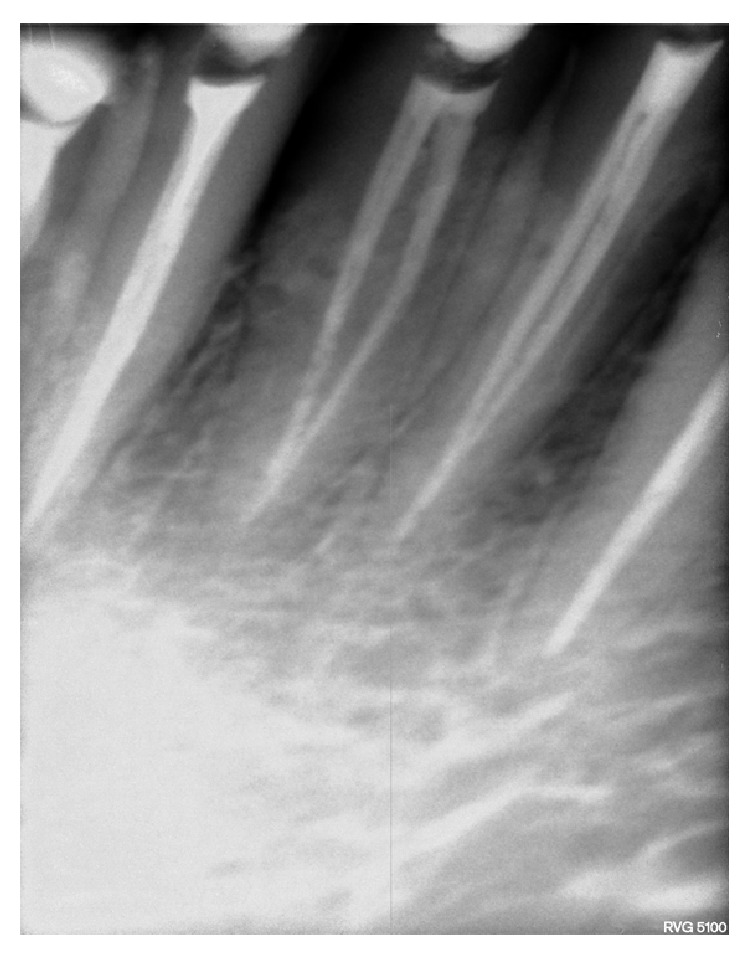
Radiograph showing obturation in two separate canals in 41 and 42.

**Figure 8:**
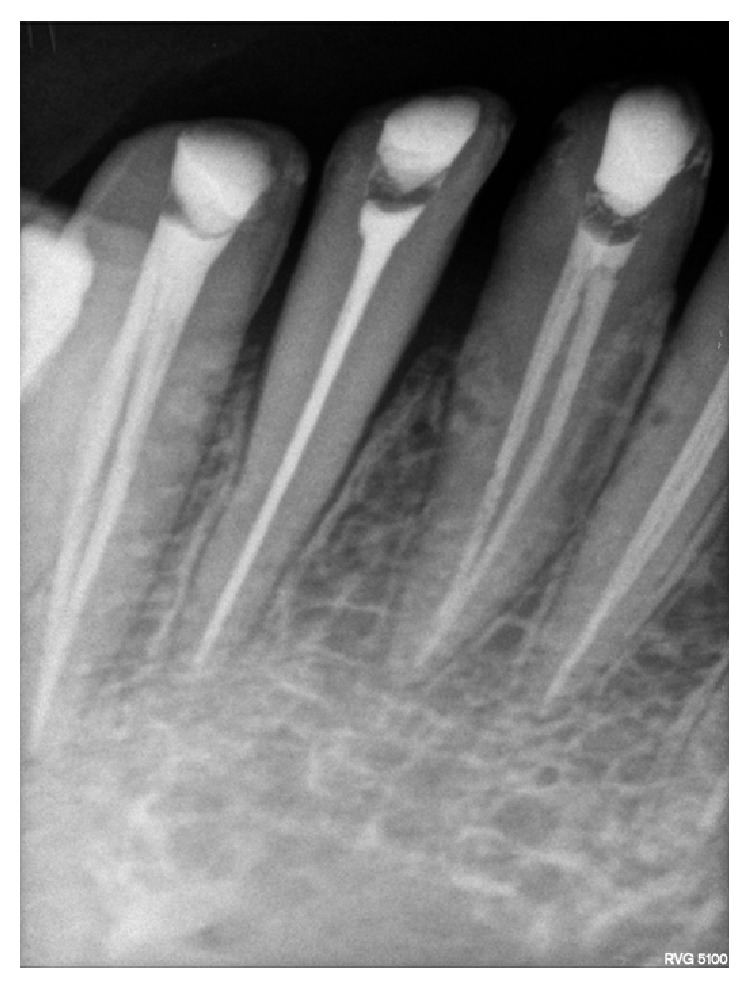
Radiograph showing obturation in two separate canals in 32 and 41.

Patient was recalled the next day (after 24 hrs), the pain and tenderness were reduced. During the next visit after 8 days; all teeth were asymptomatic and obturation was done using lateral condensation technique (Figures 6, 7, and 8). Radiographs were taken with multiple angulations (20 degree right and 30 degree left horizontal beam angulation) for better identification of two canals [5].

## 3. Discussion

A well-designed access preparation is essential for a good endodontic result. Without adequate access, instruments and materials become difficult to handle properly in the highly complex and variable root canal system. Proper access cavity preparation provides straight or direct line access to the apical foramina or at least to the initial curvature of canal to locate all root canal orifices and it also conserve sound tooth structure [6].

Mandibular incisors because of their small size and internal anatomy may be most difficult access cavities to prepare. Complete removal of the lingual shoulder is critical, because these teeth often have two canals that are buccolingually oriented and lingual canal most often is missed. To avoid missing this canal, the clinician should extend the access preparation well into cingulum gingivally, which, if present, is located directly beneath it [6]. When there are two canals, the buccal canal is the easiest to locate and is generally straighter than the lingual canal, which is often shielded by lingual shelf [7]. In this case, extension of the access opening lingually beneath the cingulum revealed the missed lingual canals in all mandibular incisors.

In this case, the private practitioner whom patient visited earlier was not well aware about root canal morphology of lower anterior or he/she must have been unable to detect the presence of second root canal, which is the main reason why the pain was possibly in lower anterior, even after the patient visited that dentist so many times. One of the main reasons for endodontic treatment failure in mandibular incisor teeth is the failure to locate, debride, and obturate the missed lingual canal. Immediate relief of pain after location and debridement of second canal confirmed the reason of pain to be the missed lingual canals.

Thus, careful interpretation of the radiographic feature taken from different angles should be done before starting endodontic treatment. One must be careful while access opening, and initial buccolingual widening of mandibular incisors and gingival extension beneath the cingulum must be made to search for a possible second canal lingually.

## 4. Summary/Conclusion

The main reason for failure of endodontic treatment of mandibular incisor can be due to the inability to detect and treat second root canal, mostly lingual canal. Thus, careful interpretation of radiographs taken from different angulations is essential. Practice of extension of access cavity buccolingually and gingivally beneath cingulum will help to detect additional lingual canal if present in each mandibular incisor.
